# Complexity in the interpretation and application of multiple guidelines for thyroid nodules: the need for coordinated recommendations for “small” lesions

**DOI:** 10.1007/s11154-025-09950-z

**Published:** 2025-02-04

**Authors:** Pierpaolo Trimboli

**Affiliations:** 1 Thyroid Unit, Clinic of Endocrinology and Diabetology, Ente Ospedaliero Cantonale, 6500 Bellinzona, Switzerland; 2 Faculty of Biomedical Sciences, Università Della Svizzera Italiana, 6900 Lugano, Switzerland

**Keywords:** Thyroid nodule, Guidelines, TIRADS, FNA cytology, Thermal ablation

## Abstract

Multiple guidelines for thyroid nodule management have been developed by endocrinologists, often in collaboration with surgeons and radiologists. While there is now a lot of scientific information available to meet the needs of healthcare providers, there is not always uniformity and standardization among recommendations. Consequently, the interpretation and application of guidelines in clinical practice remain somewhat limited. In this context, the management of “small” thyroid nodule warrants full discussion. Looking at treatment guidelines, surgery is the first-line option and the risk of cancer relapse can be assessed only after at least thyroidectomy; in addition, according to guidelines of minimally invasive treatment, thermal ablation may be considered for patients with small classical papillary carcinoma. However, the Thyroid Imaging Reporting And Data Systems do not recommend biopsy in nodules less than 1 cm; and performing biopsy may yield a result that is suspicious or consistent with malignancy without specifying the cancer subtype. With these premises, facing cases of “small” nodule less than 1 cm is challenging. Even if the recommendations of guidelines sound singularly appropriate, they may seem conflicting. Coordinated guidelines are needed.

## Context

Literature in modern medicine largely focuses on guidelines, with many original articles aiming to challenge these recommendations. Over the last two decades, there has been a significant increase in the dissemination of medical information globally, leading to a need for standardization in diagnostic and therapeutic approaches by medical professionals and patients [1]. The field of thyroid disorders is not an exception to this trend. Endocrinology and thyroid-focused societies have issued numerous guidelines on various thyroid disorders, including hypo- and hyperthyroidism, management of thyroid dysfunction during pregnancy, and particularly thyroid nodules (TNs) and cancer [2–7]. Multiple guidelines for TN management have been developed by endocrinologists, often in collaboration with surgeons and radiologists. While there is now more scientific information available for TN management to meet the needs of healthcare providers and patients, there is not always uniformity and standardization among the recommendations. Consequently, the interpretation and application of guidelines in clinical practice remain somewhat limited. In this context, the management of “small” TNs is of particular importance and warrants full discussion.

## What do we know about TNs?

TNs have garnered significant interest over the past two to three decades due to their high prevalence in the general population. The widespread use of ultrasound (US) for neck imaging has led to an increase in the detection of TNs as more patients undergo screening. These nodules can vary in size and characteristics, ranging from large goiters to small nonpalpable lesions. While the US pattern of a TN is crucial for assessing the risk of malignancy (RoM), the size of the nodule alone is not a reliable indicator. The overall rate of thyroid cancer, particularly papillary carcinoma (PTC), in patients with TNs is less than 3%–5%. Given the generally favorable prognosis of PTC and its lower frequency among patients with TNs, population screening with neck US is not recommended by WHO.

## What do we know about “small” TNs?

The US RoM assessment of TNs is independent of their dimension. For example, two TNs with major diameters of 8 mm and 35 mm, both exhibiting strong hypoechogenicity and microcalcifications, are assessed as high risk regardless of their size disparity. However, smaller PTCs have a lower potential for relapse after treatment. Therefore, the clinical significance of detecting small PTCs is generally considered to be low. Consequently, a different approach may be warranted for “small” TNs compared to larger ones. While there is no specific size threshold to define “small” TNs, those nonpalpable and measuring less than 1 cm on US are typically classified as “small”. In addition, the term papillary thyroid microcarcinoma (PTMC) was largely used to describe small, indolent cancers with a histological diameter of less than 1 cm (i.e., pT1a pN0/pNx/cN0). However, remarkably, PTMC is no longer considered as a unique PTC subtype and a (sub)classification based on its morphology rather than mere tumor size is warranted [8].

## What guidelines recommend for TN management

Various clinical, radiological, and treatment guidelines provide recommendations for the management of TNs and cancer.

1. The Thyroid Imaging Reporting And Data Systems (TIRADSs) have emerged as crucial tools for risk stratification of TNs detected on US [9–11]. TIRADSs are based on extensive data collected over the past two decades on US assessment of TNs. The primary goal of TIRADSs is to standardize the terminology used in thyroid US reports and minimize unnecessary fine needle aspiration (FNA), such as those resulting in benign/nonneoplastic cytology. TIRADSs use nodule size as a criterion for recommending FNA; smaller nodules require a higher TIRADS category to warrant FNA. Generally, TNs with major diameters of less than 1 cm on US are recommended for surveillance, with exceptions for subcapsular nodules or those with suspicious metastatic neck lymph nodes. Basically, it is advised not to perform FNA on non-subcapsular TNs classified as cT1a cN0, even if they are considered high risk based on TIRADS.
2. Since the 1980s, FNA has been recognized as the most accurate and cost-effective method for evaluating TNs, and it remains the gold standard for preoperative diagnosis of thyroid malignancy. Cytological evaluation can effectively distinguish between malignant and benign lesions, although inconclusive specimens remain a challenge [12]. Among differentiated thyroid carcinomas (DTCs), PTC is well detected on FNA samples, while follicular carcinoma is typically cytologically indeterminate. Medullary carcinoma is diagnosed or suspected in just over 50% of cases based on cytology, and other malignancies such as undifferentiated and lymphoma may require larger core needle biopsies for diagnosis [13]. It is worth noting that in most malignant cases we can only suspect the malignant type, while the subtype is seldom described in the report (e.g., “papillary thyroid carcinoma, favor tall cell variant”). Remarkably, the definition of cancer subtype/variant is cytologically unnecessary and, in any case, rarely possible or reliable [12].
3. The treatment of DTC can vary based on specific features. The American Thyroid Association guidelines recommend classifying DTCs as low-, intermediate-, and high-risk, a terminology that has rapidly and universally spread in clinical practice [6, 7, 14]. First, this 3-point system is specific to DTC and does not apply to medullary carcinoma. Second, the term “risk” refers to the probability of recurrence after treatment, typically at least surgery. Third, risk assessment is based on postoperative characteristics such as TNM staging, histological features, and imaging data. Fourth, the risk assessment helps determine the need for radioiodine treatment and tailor postoperative management for DTC. Therefore, DTCs cannot be classified according to the American Thyroid Association risk stratification system before surgery.
4. Developing reliable alternatives to surgical treatment is a significant challenge for thyroidologists. In this context, active surveillance (AS) and thermal ablation (TA) are the most frequently debated options. Both AS and TA represent intriguing challenges that warrant long-term studies, ideally randomized and controlled trials. While there is no specific international document on AS, guidelines for TA are available. One document is mainly dedicated to benign thyroid lesions [15], and a second focuses on minimally invasive treatment of malignant thyroid lesions [16]. In summary, according to guidelines, TA may be considered in patients with “classical variant PTMC” assessed as “low-risk” who refuse surgery or have contraindications for surgery.

## Major divergent recommendations in “small” TNs

At first glance, the recommendations may appear conflicting. A clinical vignette could be beneficial in addressing the discrepancies between major guidelines when applied in real-life clinical practice.

“An otherwise healthy young patient has recently received the incidental diagnosis of a “small” TN during a neck US evaluation performed for a nonthyroidal indication. Upon thyroid-dedicated US re-evaluation, the lesion is found to be sufficiently far from the gland capsule, has a diameter of 9 mm, and is classified as high risk for malignancy according to TIRADSs”.

The decision-making process in such cases is complex.

TIRADSs do not recommend FNA and suggest follow-up instead. However, performing FNA may yield a result that is suspicious or consistent with malignancy without specifying the cancer subtype. This leaves the lesion highly suspicious but without definitive proof of cancer type or aggressiveness. Before surgery, despite indications pointing toward a cT1 cN0 class, the case cannot be definitely labeled as “low-risk.” In addition, the term “PTMC” may be misleading as it typically refers to histologically proven PTC with need for its subclassification according to histo-morphological features [8].

When considering treatment recommendations for thyroid cancer, guidelines suggest surgery for both suspicious and consistent malignancy reports. It is also recommended to thoroughly evaluate the patient’s demographics, individual risk factors, sonographic RoM, patient preferences, and mutational status if available. Future studies are needed to better understand the role of molecular tests in discriminating small PTC with indolent behavior from other types. The guidelines recommend using a three-tiered (i.e., low-, intermediate-, and high-risk) recurrence risk stratification system for patients with DTC who undergo thyroidectomy. According to guidelines, minimally invasive treatments, such as TA, may be considered for patients at high surgical risk, with a short life expectancy, other significant comorbidities, or who decline surgery or AS.

**Fig. 1:**
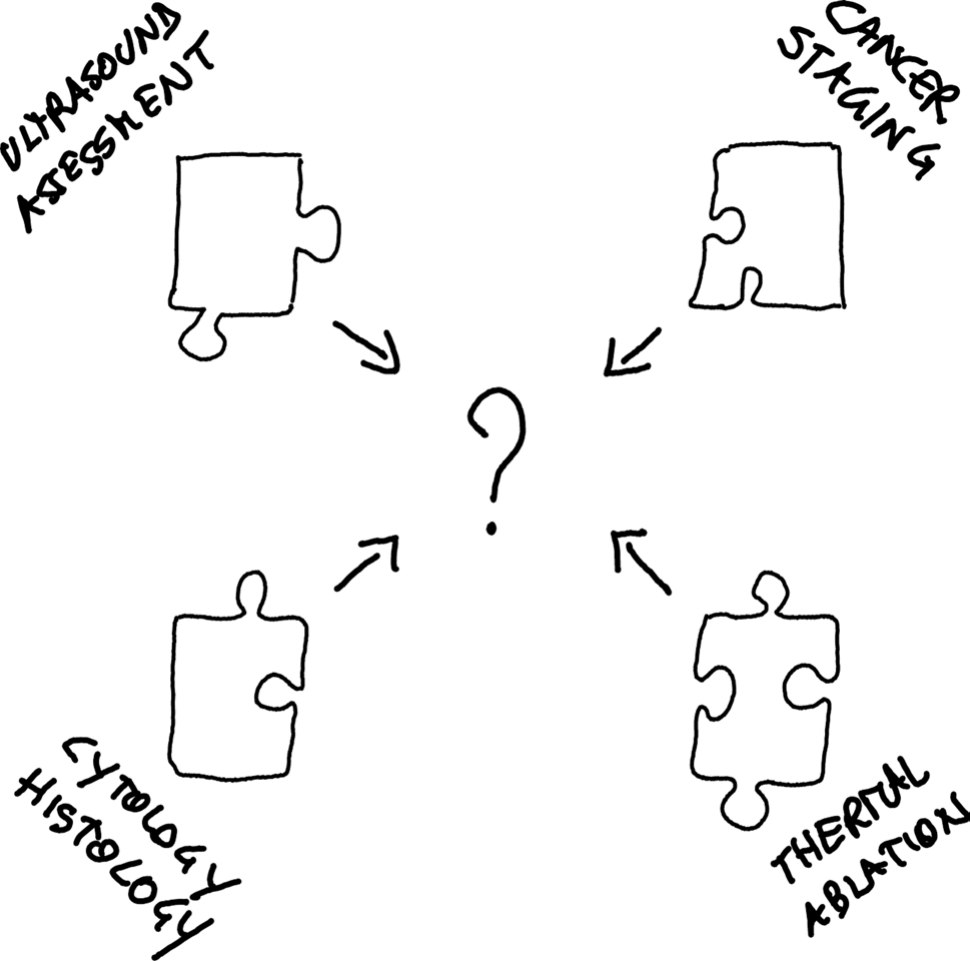
The impossible puzzle of guidelines recommendations for managing “small” TN

Figure 1 schematically illustrates the discrepancies in guidelines recommendations as an impossible puzzle, highlighting conflicting advice that can make overall understanding and their integrated application challenging.

With all these considerations, discussing treatment options with patients can be challenging due to technical terminology, divergent recommendations, and the potential unavailability of testing. All the above recommendations are singularly reliable. However, patients may have several questions, such as why a nodule should be treated despite its low aggressiveness and lack of indication for FNA; how nonsurgical options can be considered before malignancy is confirmed, especially given the limited reliability of cancer subtyping; how suspected cancer can be staged without thyroidectomy and its risk of recurrence assessed; and what follow-up strategy should be implemented if surgery is not performed. Clear and convincing answers to these questions are currently lacking. In addition, multidisciplinary discussions among endocrinologists, surgeons, pathologists, and nuclear medicine physicians may be complex and influenced by factors such as the local health system, cost-effectiveness, patient preferences, institutional context, surgeon expertise, and potential ethics committee approval of nonsurgical strategies.

## Hopeful sight of next TN guidelines

Guidelines will remain pivotal for providing continuous education and guaranteeing high-quality clinical management of TN patients; and societies will continue to furnish their members and delegates with recommendations to standardize and improve their clinical practice. As the incidence of TN in the general population is very high, and considering that the TN diagnosis is frequently incidental with initial assessment charged by general practitioners, steering committees of various societies should activate an inter-society dialogue to pave the way for developing coordinated recommendations, at least about “small” TN. Examples already exist, such as the joint statement about radioiodine therapy of DTC [17] that addresses controversies associated with the therapeutic uses, the consensus about levothyroxine/liothyronine combination to treat hypothyroidism [18] that summarizes the evidences achieved in this area, and, more recently, the expert consensus on lexicon to use for reporting US appearance of TNs [19] presenting the results of the first phase of the important project to develop an international joint TIRADS that will replace the current ones. In these cases, the societies that had already published documents in the area of interest 1) identified points of disagreement between documents, 2) agreed to participate in a joint document to summarize controversies and harmonize the recommendations, 3) appointed their representative committee, 4) supervised the work of the multi-society group, and 5) endorsed the final paper. Such a process represents a model that guarantees each society to argue its previous proposals with the intent to achieve mutual consensus. A joint inter-society document about small TN management would provide generalizable indications for clinical practice to obtain uniformity between clinicians and then enhanced patient care.

## Conclusions

Singularly, the recommendations of current guidelines to manage TNs appear reliable and sound appropriate. However, they may seem conflicting. Medical guidelines are essential tools for improving clinical practice and enhancing patient care and quality of life. The importance of guidelines cannot be overstated, as they provide a framework for evidence-based decision-making. However, guidelines can be compared to trees, with an initial trunk that branches out over time, leading to potential overlapping and confusion. To prevent guidelines from becoming a tangled forest, it is crucial to ensure coordination and consistency among them. Furthermore, attention to the terminology used in guidelines is also critical for clarity and effective communication [17, 19].

## Data Availability

No datasets were generated or analysed during the current study.

## Abbreviations

AS: Active surveillance
DTC: Differentiated thyroid cancer
FNA: Fine needle aspiration
RoM: Risk of malignancy
TA: Thermal ablation
TIRADS: Thyroid Imaging Reporting and Data Systems
TN: Thyroid nodules
US: Ultrasound
